# Case of Filariasis with Abscess
1Read at the meeting of the Bristol Medico-Chirurgical Society, January 8th, 1908.


**Published:** 1908-06

**Authors:** A. L. Flemming

**Affiliations:** Anæsthetist, Bristol Royal Infirmary


### CASE OF FILARIASIS WITH ABSCESS.[1]

BY

A. L. Flemming,

*Anæsthetist, Bristol Royal Infirmary.*

The patient, a man aged 30, came under observation on the day following his arrival in England from Duala, in German West Africa, after spending a term of six years in that country, during the latter three years of which period he had been subject to Calabar swellings. He had never contracted syphilis.

*Onset.*—He was in good health when he left Duala, on November 24th, 1906, but when two days out from Madeira, on December 13th, he was suddenly seized with a severe pain in the back, which he could not explain in any way. From this date onward the pain was incessant, until the patient arrived in Clifton, four days later.

*5th day of illness.*—When first seen he complained of pain in the back so acute that he resented the slightest movement. He lay perfectly still on his left side, his aspect was that of severe illness, with anæmia and furred tongue. The skin was universally

[1] Read at the meeting of the Bristol Medico-Chirurgical Society, January 8th, 1908.

pigmented and studded with small scars left by boils and craw-craw ulcers; the left arm presented one of these ulcers not yet healed. Examination of chest and abdomen revealed nothing abnormal, with the exception of impaired resonance and deficient breath sounds at the left base, with no adventitious sounds. Temperature, 98; pulse, 84; respiration, 20; urine, 1022, acid, no albumen or sugar. The back showed an almost imperceptible fulness over—and apparently of—the right erector spinæ at the level of the first lumbar spine. This swelling was tender, but neither hot, red, fluctuant, nor œdematous. Treatment: Aspirin, mesotan, hypodermic of morphia, and an aperient.

*9th day.*—General condition worse, malaise increasing; abdomen tense, occasional vomiting, inability to sleep; the swelling larger, measuring five by two inches, with also painful spasms of some fibres of external oblique to inner side of rt. ant. sup. spine of ilium. Temperature, 99; pulse, 90; respirations, 24; blood count—reds 4.5 millions, whites 8,000, hæmoglobin 75 per cent.; differential count—polymorphonuclears 66, lymphocytes 16, hyalines 8; *eosinophiles* 10 *per cent.*

*13th day.*—Condition more grave. Swelling of back six by three inches, with long axis parallel with spine. Marked dulness of left chest from base up to angle of scapula, with absence of inspiratory and very weak expiratory sounds. Apex beat displaced to ½-inch outside nipple line, in fifth space.

*15th day.*—Swelling measured nine by five inches; no redness or fluctuation. Examination of chest revealed friction sounds, chiefly expiratory, over liver in front and in axillary region, also in left axillary region over lower interspaces. Air entry at both bases very deficient. Apex beat 1½ inches outside nipple line. Temperature, 100; pulse, 84; respirations, 22; blood—whites 10,000, eosinophiles 18 per cent.

*18th day.*—Anæsthetic administered, and the swelling was incised. An exploring needle was inserted in several directions, and a portion of muscle removed and sectionised, but all with negative results.

*27th day.*—The peripheral blood was found to contain filaria, the average number in each drop examined being ten by day and only three or four at night. The physical signs of the chest were so suggestive of an accumulation of fluid that an X-ray screen was used, but it failed to reveal any collection. During the past ten days Calabar swellings had appeared on the abdomen and left wrist, measuring about $1\frac{1}{2}$ inches, and lasting for twenty-four or thirty-six hours in each case. Blood count showed: whites, 10,000; eosinophiles, 22 per cent.

40*th day*.—The general condition improved, and the swelling smaller—six by four inches.

61*st day*.—Swelling now measured only four by two inches, but during past three weeks there had been two acute exacerbations, with sudden increase in size of swelling and in severity of pain and general symptoms, lasting for six or seven days.

66*th day*.—Under an anæsthetic the tumour was incised, and an abscess was found situate in the deepest layer of muscles to the right of the spine. It tracked between the muscle fibres, and contained 3 oz. of thick, fusty-smelling pus. The cavity was lined with a thick layer of inspissated pus and lymph; this having been curetted away, the cavity was drained and the skin sutured. The contents of the abscess were carefully examined for the dead parent, Filaria Loa, but this, as might be expected from the character of the abscess contents, was not found.

77*th day*.—The operation wound had healed, and the patient was free from pain, feeling well, and the physical signs of the chest were normal, the apex beat being inside the nipple line and the air entry being good at both bases. The blood count now gave: reds, 4.500000; whites, 8,000; eosinophiles, 16 per cent. Filaria: by day eight in each drop; at night, *i.e.* from 10 p.m. till 8 a.m., none.

A few weeks later patient returned to Africa, and nine months later his left eye became "inflamed" for two days, during which time he twice saw a worm "under the skin of the eyeball."

Some points of interest in the case were the following:—

*The physical signs*.—The dulness of the left chest, with almost absent breath sounds and the displacement of the apex beat to $1\frac{1}{2}$ inches outside the nipple line, seemed to be due, in a large measure, to the patient lying persistently on his left side day and night. The friction sounds on the left side, which developed after two weeks' illness, suggest that the inflammatory process around the abscess spread through the diaphragm to the pleura.

*The blood-count* afforded the most obvious clue to the presence of some parasitic infection, inasmuch as the eosinophiles were always in great excess.

*The identification of the Filaria* was rendered difficult by their appearance in the peripheral blood at night as well as by day. In all other respects—in their shape, size and structure—they resembled the F. Loa; but that their periodicity should be upset by the habits of the patient was not in accordance with the recognised habits of this species of Filaria, and the possibility of a double infection with F. Loa and F. Bancrofti was considered. But the night blood never contained so large a number as the day blood, and as soon as the patient resumed his normal habits the night blood was quite free of Filaria, which pointed to the diagnosis of F. Loa being the correct one.

*The abscess.*—In many reported cases the parent worms have been recovered from abscesses, but as the mature parasite measures only 30 mm. by .3 mm., and as this abscess had existed for sixty-six days before it was evacuated, and its contents had undergone considerable inspissation, it would be improbable that the parasite, if dead, would not be disintegrated. The abscess contents possessed a peculiar fusty odour.

I am much indebted for valuable help given to me by Sir Patrick Manson, Mr. T. Carwardine, Dr. Charles, and Mr. James Taylor.

POSTSCRIPT, May 29th, 1908.—I have this day had an opportunity of seeing the patient, who has returned from Africa. His day blood contains numerous filariæ, 14 and 16 in each drop examined; whereas the blood at midnight shows no filariæ.—A. L. F.

